# On Impulsive Insanity

**Published:** 1848-10-01

**Authors:** 


### ON IMPULSIVE INSANITY.

Few positions in life are more harassing than that in which the physician is placed when called before the tribunals of justice to offer testimony on the state of mind of an individual accused of a heinous crime, in mitigation of which insanity is pleaded. Whatever the conviction may be on his own mind, he has serious difficulties to contend with, if he attempt to combat the prejudices and the ignorance of those by whom he is surrounded; they, for the most part, boasting of their yielding only to common sense, forget that the circumstances relative to which they are to assist in forming a judgment are the exceptions to the general laws by which human nature is guided, and that they can only be elucidated by facts of an extraordinary character, which rarely present themselves in the state of society in which the individual exists. The attempt, by comparison with what he has already seen, known, or read, to explain the views that he entertains, is liable to be considered either as the offspring of a mawkish sensibility, or, what is still worse, the sordid result of the paltry remuneration which, as a physician, is awarded to him. He has to encounter the sarcastic doubts, the suspicious hints of the cold and calculating advocate, the unyielding dogmas of the judge, and the matter-of-fact preconception of the jury. He feels that there is no corresponding impulse in the minds of any of those before whom he stands, and that he is not only in opposition to common prejudices, but that he has to encounter the specious plausibility which wraps itself up in the appeal to the popular feelings and passions. The individual arraigned before the laws of the country has exhibited no manifestation of mental disease which has led to the commission of the act for which he is tried; there have been no antecedent circumstances so strongly marked as at once to betray the latent malady, and therefore is he a criminal, and any attempt to shield him from punishment is almost as great a crime as that of which he has been guilty. Yet what an infinite variety of alterations in the intellectual system are there which may impel the victim to irresistible actions which, according to the law, divine and human, are the common result of guilt. That which adds to the perplexity under which the medical man labours, is the absence, until within a very few years, of those decided landmarks which are the guide by which every one must steer his way, and even as yet there remains so much to be done to elucidate the subject of impulsive insanity, that he cannot yet feel himself on safe grounds, but must look with anxious attention for every fresh means offered to him of smoothing the difficult path on which he has entered. In later days, very little has been done which throws any fresh light upon those fearful paroxysms which have occasionally suddenly burst forth and led to the commission of every horror that swells the catalogue of human crimes. Scarcely, indeed, will the world at large recognise that which constant experience has shown to the student of psychology—and however high he may be placed, so as to look down upon the ordinary race of beings, their failings and their irregularities, the judge himself too often fails in understanding the strong evidence of one whose life has been almost passed amongst the beings whose cause he has to elucidate.

In examining into the nature of instinctive madness, it will be found that persons are seized with an uncontrollable desire to the commission of acts, some of ferocity, others of cunning, others of depravity, which, from the utter impossibility of their ever deriving any advantage to themselves, can only spring from a morbid perversion of the feelings, the emotions, the passions. There can be no cause traced for the sudden feeling which has taken possession of the mind. Beings harmless, innocent of evil, have with inconceivable rapidity rushed upon the commission of acts whose tendency they seem scarcely to know, and have without remorse murdered the beings they have most deeply loved. The first portion of this vast subject which is to be studied is, how far the plea of insanity is to be admitted in extenuation of acts resulting in death; and under this head are to be considered, suicide, homicide, infanticide.

The first of these points has varied as a criminal act, according to the religion and morality of different nations, and has not only been tolerated, but applauded, under different conditions, and even the same individual, Madame de Staël, has expressed opposite opinions at various eras of her life. The first time we hear of it as a question before a criminal court, in modern days, is in the year 1744; when an individual in the province of Artois, of the name of François des Bureaux, escaping from the keepers in whose care he had been placed, hung himself in his granary; but his wife discovering his flight, rushed after him, and succeeded in cutting him down and restoring him to animation. She attempted, as she had once done before, to have him proved insane; but in this she was unsuccessful. He was seized, and tried for this attempt, was condemned to the galleys, and his property confiscated. An appeal was made to the council at Artois, who, with greater justice than the first tribunal, pronounced, upon an inquiry into his state, that he was insane, and remitted the previous sentence. Upon this his wife applied for a curator, to take charge of his person and property; but here again the local court denied equity. In the meantime, the brother-in-law of the insane man, who was the lieutenant-justiciary of the village, taking advantage of his legal position, deprived the unfortunate Des Bureaux of his property, by making him sign various deeds which conveyed the property to himself. In 1768, the wife, when it was too late, obtained a sentence of interdiction, and of nomination of a curator for her unfortunate husband, who died shortly afterwards, in a state of the greatest misery and of decided insanity. His widow then sought from the council of Artois the annulment of all acts done by her husband since the period when that tribunal had declared him insane; thinking, with the rest of the world, that if he were found insane by the criminal court, the same circumstance would occur in the civil court. But she was mistaken; the cause was given against her: it remains to this day amongst those celebrated trials which have been collected together, and are proofs of the law's delays, its uncertainties, and its ambiguities. In 1777, the question, whether suicide was a proof of madness, was brought before the parliament of Paris; but there the same fate attended it—

the cleverness of the advocates stifled the prosecution of the inquiry. The opinion of Foderè, which has not been fairly quoted by some of the English writers on the subject of suicide, was that suicide was always the result of mental aberration; that it was the result of madness existing at some time anterior to its commission,—a temporary delirium, produced by the momentary violence of one of the passions,—or that it was a delusion of the mind and of the judgment. He observed, that all those who perished by their own hands had, at some period or other, been affected with insanity, either disclosed or latent; that they were, from certain conditions of the sensorium, either predisposed to an access of melancholy, or to sudden paroxysms of rage; that there was always a certain temperament in which this morbid state was more generally developed. It was not the lively, the gay, the animated, who terminated their own existence, but the melancholy and the morose. To this it is to be answered, that the happy have no reason to wish to leave the scene of their enjoyment; but those to whom fortune is niggard of her gifts are, of course, less anxious to stay, "uninvited guests at Nature's great feast." He points out that a disordered state of the brain will induce the irresistible desire; in which he is borne out by more modern authors. He thinks that examination of the dead body will show, either the vessels of the brain distended, gorged, as if injected with blood, organic malconformation of the cranium, and different lesions of the organic viscera. This humane opinion, given by the great master of forensic medicine, soon became the foundation on which both lawyers and the members of the medical profession throughout Europe built their own structure; and Avenbrugger of Vienna, Le Roy of Antwerp, and Noast of Leyden, following up his views, showed how frequently suicide depended upon physical causes; and whilst studying what were the causes, succeeded in restoring some individuals who were previously lost to society. In the day in which Foderè wrote, he lamented that suicide was so common in England, and expatiated with considerable feeling on the necessity of legislating on the subject; and pointed out that at Marseilles, where there was almost an epidemic mania for suicide amongst the young females, it was checked by the announcement that their dead bodies should be stripped, and exposed to view. Had the learned jurist lived at the present day, he would have had the regret to find that the number of suicides in France has increased to an enormous amount; that, owing to the impression made upon their minds by some of the fashionable novelists of the day, young people of ardent enthusiasm now destroy themselves on the slightest occasion. Love, disappointment, losses at the gambling table, are now the constant causes of self-destruction. The ordinary termination of a romance is the suicide of two or three of the principal persons; indeed, the catastrophe would scarcely be complete without it; and the most fascinating female writer of the day, who has lately as much distinguished herself for her political as for her imaginative writings, actually sends the hero and heroine of one of her tales to Teneriffe, to throw themselves from the peak, whilst the sang froid with which she discusses the question quite equals that with which Foderè wrote his opinion on the Englishman's malady. Homicidal madness has only within a very few years attracted the attention of writers on psychology and physicians; indeed, until the year 1826, when Georget published his "Medico-legal discussion upon madness, or mental alienation, followed by an examination of the criminal trial of Henriette Cornier, and of many other trials in which that malady has been alleged as a cause for defence." Whatever may have been the opinion of mankind as to the madness of numerous enthusiasts who have been guilty of murder, the subject has not till lately been boldly advocated as an excuse for the crime. The assassination of the Duke of Buckingham, in the days of Charles I., was viewed with horror, and was universally acknowledged to be the mad act of an insane person. The murderers of Henry III. and of Henry IV. of France have been supposed to come under the same catalogue; yet no one was found to plead it as a bar to their execution, or in mitigation of the ferocious tortures which Ravaillac had to undergo. The age was not sufficiently advanced in the knowledge of mental disease, nor was there the same spirit of bold humanity, which has, in defiance of the difficulties, attempted to establish the truth, and to plead for the unfortunate beings.

Although the case of Henriette Cornier, so admirably discussed by Georget, is thoroughly known to most of those who have studied medical jurisprudence, yet as references must occasionally be made to it in the succeeding pages, and as upon this case so much of the history of homicidal impulse rests, it must be briefly narrated here. Henriette Cornier was a young girl of about twenty-seven years of age; she was of a mild and lively temper, cheerful and gay, and it had always been remarked that she was much attached to young children. In the month of June those who were immediately about her appear to have been struck with a singular change that came over her. She was no longer full of vivacity and playfulness; she became taciturn, melancholy, and apparently always plunged in a deep reverie. She was discharged from the situation in which she had for some time lived. She gradually was absorbed in thought, and at last became overwhelmed with a degree of stupor. The friends who were in immediate communication with her began to suspect something amiss; they imagined that she had become pregnant, but soon found their mistake; they in vain tried to obtain from her some reason for this remarkable change. They frequently asked her what was the cause of this dejection, but she remained deaf to their inquiries, whilst her state of stupor increased. In the month of September she attempted to put a period to her existence by drowning herself in the Seine. The solicitude of her friends increased, and in the following month they succeeded in obtaining for her a situation in the family of Madame Fournier. Here she exhibited much the same state of mind; there was no diminution in her state of dejection, and she was under the influence of precisely the same melancholy. It was on the 4th of November that the act for which she was brought before the tribunal occurred. In the afternoon Madame Fournier went out, desiring Henriette to go to a shop in the neighbourhood, kept by the Dame Belon, and purchase some cheese. In this shop there was a beautiful little child, the daughter of Madame Belon, about nineteen months old, of whom Henriette had always seemed to be very fond, caressing and nursing it. On this occasion she displayed the same feeling, and entreated the mother to let her take it a short walk, to which she unwillingly consented. Henriette then took the child with her into the house of her mistress, where there was no one, carried her up stairs, taking with her a large knife, with which, after laying the child on the bed, she cut off her head at one stroke. She laid the body on the floor, and placed the head near it by the window. A quarter of an hour elapsed, during which Henriette betrayed no emotion whatever. The mother came to the house, and, from the bottom of the staircase, called to her. She replied by asking her what she wanted. "Your child is dead," said Henriette, without betraying again any emotion. Belon, frightened, was louder in her inquiry, to which Henriette made the same reply, "Your child is dead." As the frantic mother rushed up stairs into the room, Henriette took up the head of the child, and dashed it into the street. The mother ran into the street, overcome with horror. When the father and the police arrived, they found Henriette sitting perfectly composed, near the decapitated body, gazing at it; her clothes were saturated with blood, whilst in her hand she held the bloody instrument she had so lately used. She did not hesitate to avow herself to be the authoress of the deed, and seemed to dwell with pleasure on the premeditation which led her to it, and stated that she had caressed the child with the hope of deceiving the mother, and leading her to place her under her care. In vain was it attempted to make her acknowledge some sorrow. She showed not the slightest feeling; and when urged to give a reason for such an act, she only said, " I determined to kill it."

The excitement that followed upon this melancholy homicide was great. When the case came on for trial, the plea advanced was insanity. The court appointed Esquirol, Adelon, and Levelle to visit her, and to make their report upon her state. They visited her; they could discover no proof of actual insanity, but they would not express an opinion that it did not exist. She was placed in the Salpêtrière; there she was frequently seen by the physicians from February to June. They report that they merely observe defection of mind, slowness in the manifestation of thought, and profound grief. These, however, are explained by circumstances, and are no proof of derangement; that the opinion as to the question of her insanity is influenced materially by her previous history. If the allegation be proved, that long previous to her act, her habits, her whole character had been changed, that she had become at a particular period dejected, gloomy, taciturn, restless, prone to reverie, and had occasionally attempted suicide, it would appear that her present state is not the result of existing circumstances, since it has lasted a year before the commission of the act, in which case the opinion of her insanity would be materially influenced. The trial came on again, exciting throughout the whole of France much interest; but the opinion generally expressed being that Henriette was guilty of a most atrocious murder, which no attempt of lawyers and medical men should screen from condign punishment. The procureur-general treated the opinions of Esquirol, and the opinions of the physicians, who were generally inclined to view it as a case of monomania, with the utmost disdain. The jury brought in a verdict of voluntary homicide, without premeditation; and the sentence was, that she should be perpetually imprisoned, with hard labour, and be branded. She heard the sentence with the utmost indifference. This may be considered as the first step made in the behalf of those who are driven by insanity to the commission of crime. That the opinions of the physicians should be treated with the greatest severity, that the judges should hear the new doctrine with a repugnance amounting to disgust, cannot astonish those who have since watched the march of public opinion. M. Georget, especially, met with the fate of all new innovators; the public ridiculed him, as it did afterwards Dr. Haslam in England, when he declared that he recognised a madman by his smell; and they treated him much in the same way as has done the English public those physicians who have from time to time, preferring truth to popular clamour, asserted the incapability of reasoning of some of those who have been brought up as culprits. But there was a still more extraordinary influence produced by this discussion and the sensation produced. Many females fancied that they felt an irresistible impulse to the commission of a similar act. No less than six cases came under the immediate care of Esquirol. In England, too, it has been found that where instances of impulsive insanity have been the subject of public notoriety, imitators have quickly sprung up; where attempts at destruction have been made, it has been observed that the example has been quickly followed, and the contagion has spread.

In the greater number of cases which come before the public tribunals, upon a witness being examined as to the previous state of the individual accused of the commission of a crime whilst in a state of aberration of mind, he will remember that the first coming on of the unusual behaviour was marked by stupidity, and by a species of gloom. It was with difficulty that the party accused was made to understand what was said to him, he being in a state of stupor. Authors have generally characterised this stupidity—more especially Esquirol and Georget—as a suspension, or at least an embarrassment of ideas. Others think that the intellectual faculties are weakened, or even entirely suspended. Some look at this stupidity as the abolition, or rather the rapid suspension, without fever, of all the cerebral faculties. It has remained, however, for Baillarger very lately to point out the true nature of the stupidity attended with melancholy. He has shown that the invalid is so thoroughly occupied with internal illusions and hallucinations, that he is completely shut out of the external world; absorbed within himself, he is most probably brooding over the act he is about to commit. This stupidity arises from the utter incapability of rousing himself out of the all-engrossing thought in which he is buried. He is an imaginary existence, which overwhelms every idea; he is dreaming elsewhere. Out of eight persons whom he found apparently in the extreme of stupidity, five attempted suicide. The apparent imbecility, the absence of all manifestation of the power of reasoning, led at one time to the idea that these stupid persons required little watching; the reverse has been found to be the case: they have sought, when the opportunity has presented itself, the means of self-destruction; and it is now ascertained, at the great institutions in Paris, that the stupid require by far the greatest share of attention. A young girl, who, in consequence of her accouchement, had become completely stupid, and was regarded as little likely to destroy herself, seized the moment of the absence of a guardian to accomplish her purpose. When there is a gloomy misanthropy, and a sullen refusal to join in the habits of society, there is generally one absorbing thought which swallows up all the rest; and if it be one that the individual knows to be connected with the commission of crime, it acts upon the mind till there is no other thought remaining, and everything else seems to be incapable of yielding food for a moment's reflection. It was by a close examination of convalescent patients that Baillarger arrived at the opinion, that, however stupid the patient might apparently be, that intellect was never suspended, but that a gloomy or a melancholy thought had taken such entire possession of the mind within, that external objects ceased to produce the slightest interest. There is another observation which he has lately made, which deserves to be known—viz., that hallucinations coming on between the state of sleeping and waking are often the precursory signs of an attack of madness. A German peasant woke up in the middle of the night, and struck with an axe a phantom which he saw before him. Shortly after that he killed his wife, with whom he had always lived on the best terms, in that way. Before that, he had never shown the least disposition to insanity, nor did he ever afterwards. The case was exceedingly interesting to the medical jurists, and it was the cause of a long consultation, which has been recorded by Marc. The members of the consultation declared that the murder was committed between sleeping and waking. This opinion was a most unsatisfactory one, as it led to the idea that a man might commit a murder in the night, and when found in the morning in a perfectly sane state of mind, would be allowed to plead an hallucination, and there was no foundation in any medical authority for the supposition that the insane were liable to an access of paroxysm after sleep. Thirty instances have occurred to Baillarger which prove that this phenomenon occurs. These attacks are the forerunners of insanity, especially in those predisposed to it. Any hallucinations between sleeping and waking foretel the coming on of disease: it may be months before it will show itself, but it is generally not more than two or three days.

One of the cases of stupidity associated with suicidal impulse on which Dr. Baillarger founds his views occurred at Charenton. A young man, twenty-five years of age, the head of one of the departments of the administration, had before been attacked with insanity, once at fifteen years of age, and again at twenty-two. The first had lasted six weeks, the second fifteen days only. From the information obtained from the family, it appears that he had just recovered from an attack of intermittent fever, which had lasted six weeks, when the third access of paroxysm came on, without any apparent cause, after several days of severe cephalalgia. The symptoms at the commencement were those of cerebral fever; there were convulsions, which came on at intervals for about three weeks. He was impressed with the idea of committing suicide, for he had several times attempted it with cutting instruments; he had tried to throw himself out of the window; he had swallowed money with the idea of destroying himself. This attempt had produced no bad effect. He had been bled several times, had leeches applied, and used baths. At his entrance into Charenton, he presented the following symptoms:—His complexion pale, his eyes were fixed wide open, and constantly turned towards the earth. His physiognomy has lost all animation, and presents the appearance of the most decided stupidity. He passes his time in the same spot, seated, in a complete state of dumbness, and appears ignorant of all that passes around him. If he is spoken to, it must be in a very loud voice, and questions must be repeatedly put to him in monosyllables, slowly and loudly. If he seems to wish to walk, he is afraid lest he should fall; he supports himself by leaning against the wall, or those who are near to him. His walk is very slow. The only sign of activity or of intelligence that he gives is when he opposes being sent to the bath. Often in the course of the day he gets to his bed, and lies down. His memory seems entirely gone. His stupidity is carried to such an extent, that he is obliged to be fed; his inattention to his dress is very striking; and such is his want of cleanliness, that he is compelled to have a long frock put on. His sleep is profound, his appetite good. Shortly after his arrival, a blister was ordered to the nape of his neck. He soon after complained of the bad effects, but from that moment he began to improve a little. His answers were longer, his voice stronger. He could not, he said, get rid of his ideas—they tormented him. His physiognomy still presents an air of great stupidity, and his want of cleanliness the same. Sometimes he bursts out into a fit of loud laughter, on seeing some patient dressed, like himself, in a frock. About the end of a month a change for the better was visible: the fæcal and urinary excretions were no longer involuntary. On learning that he was a musician, and he is asked to take his violin, although his mind is still very much embarrassed, he takes it, and each successive day plays for some hours. M. Baillarger left him early in the month of November, and saw him again in December: he was then completely restored to health. Instead of a stupid, insane person, he found a young man of an open, animated countenance, having much solid and varied information. He was anxious to learn from him what had been his sensations during the three months of stupidity and idiocy; and he could not have addressed himself to a person better capable of explaining the phenomena to which he had been subjected. He described it as a state of dream of intolerable length. Everything seemed transformed: he thought that there was a general destruction. The earth trembled, and opened at his feet; he was on the point of being swallowed up every moment by an abyss that was at his feet. He seized hold of persons near him, to prevent his falling into precipices, which were like the craters of a volcano. He took the hall where were the baths for the regions below. He thought that everything about him would be drowned. In his mind, at last, all became chaos, confusion; he no longer distinguished day from night; months passed like years. He then accused himself of all the misery that occurred, and then began to think of destroying himself, which he attempted to do several times. Then, the more he suffered, the happier he became; for he looked upon his sufferings as the expiation of his crimes.

The other observations and experiments which the practice of M. Baillarger has furnished him are most valuable; and he has commenced the publication of a volume of the deepest interest, but it is unfortunately interrupted by the melancholy state of his native land, and by those events which are daily adding new victims to the lunatic establishments. Nothing can be more instructive than the history which convalescent patients give of their internal sufferings, their convictions, and the line of demarcation which they themselves are able to draw between that which exists only in their own imagination and that which actually passes around them. The melancholy person is generally able to preserve his connexion with the external world; he knows his physician—his attendants—that he is in an hospital; but the stupidly insane are concentrated in themselves,—now and then they burst, as it were, out of the sepulchre in which they are entombed. Aubanel and Thore consider this to be one of the characteristics of this form of insanity; and this alternation of activity and apathy often leads to the confounding of the melancholy with the stupid. Some days they appear to have a sudden ray of reason, and again they sink into their hopeless condition; and then it is that they wish to die, to kill themselves. How long an individual may be brooding in apparent stupor, in overwhelming dejection, over some intended act, it is difficult to judge; but all authority goes to prove that, for some length of time, those who are in the immediate society of an individual thus actuated, perceive that there is something unusual in his manner, but are, from their habits of life never leading them to observation, little aware of the phenomena which will in all probability develop themselves.

The symptom which precedes an act of violence—stupidity gradually coming on—has not yet called forth much observation from medical men; yet it is one which deserves great attention: scarcely is there now a case in which maniacal homicide has been proved, but the ordinary remark of the newspaper that records the facts is, "that the individual had a remarkably sullen and stupid appearance." On questioning the witnesses as to their previous knowledge of the patient, and whether he betrayed the slightest appearance of insanity, reference is more generally made to the apparent consistency of conduct and of expression, to the coherence of thought and of speech, than to the disposition and temper; yet it is in these that the change is most remarkable; it is during the incubation of the disease that the mind, absorbed in itself, exhibits a stupidity, from its abstraction from all the world, whilst brooding over the deed which bursts forth as the sudden display of mania in its most horrible form. It will be remembered that, in the case of Henriette Cornier, she became taciturn melancholy, and that she sank into an apparent state of stupor; such, too, was the case with Mrs. Brown, tried at the Old Bailey for the murder of her husband's child, aged three years: for several weeks previously to the deed, she was described by the witnesses as being in a state of indescribable sullenness and gloom; she was almost incapable of being roused into action, and sank, when slightly roused, into the same state of lethargic torpor. In this instance, the jury was satisfied, from the testimony of those about her, that in these melancholy conditions there was sufficient to authorize them to believe that she was not in the ordinary state which would enable her to form a judgment between right and wrong, and acquitted her, accordingly, under the plea of insanity. Those who, from their infancy, are gloomy, silent, moody, and melancholy beings, are generally shunned by their fellow-beings; they carry that about them which renders them, in the eye of their fellow-creatures, dangerous. Their unsociability is felt to spring from some bad feeling lurking at their hearts; they are observed to be in constant reverie, and were the subject that absorbs them known to others, it would be found that they knew it was one which was horrible to others as well as to themselves.

In the fifth number of the "Edinburgh Law Journal," is a paper on homicidal insanity, by Mr. Simpson, well worthy perusal. The case of Papavoine, who murdered the child of a lady walking in the wood of Vincennes, and wounded another, exhibits the gradual development of insanity in one predisposed to it—the physical and moral changes that occurred, his melancholy and hypochondriac state, his sombre, suspicious feelings, his easily exasperated temper. Yet, although there could be no motive for the act, which was evidently committed during a paroxysm of insanity, he was convicted and executed as guilty of murder. In the cases which have most generally been adduced, there has been but little of irritability of temper, little waywardness of manner; but there has been that absence of all power of controlling the thoughts, which has at length led to the perpetration of some horror. It is to be borne in mind, that this state of the intellectual faculties is essentially different from that which prevents the development of the ideas in the imbecile and in the idiot; it is not that there is any incapability of the nervous system to convey healthy impressions to the brain; it is not that there is anything deficient in the organization, but that the atony and inactivity, amounting to stupidity, arise from a preoccupation of thought, which is complete and overwhelming.

There are certain indications in the state of the stupidity of the insane which have to be studied, and upon which as yet little light has been thrown. In the first place, the state of the eye is one requiring more elucidation. There are two states of incapacity of vision occasionally noticed; the one, in which the eye wanders from object to object, never remaining one instant fixed, yet seeing nothing; there is an eternal mobility, which ought to embrace everything, yet returns no impression to the brain; there is also an obstinate, indefinable fixity of the eye, which gazes with intensity, yet is there total blindness to every object: the hearing is apparently lost; no sound seems to be transmitted that awakens any feeling of pleasure, yet when words are frequently repeated, they seem at last to be heard and appreciated. The faculty of rousing the listening power, which seems to be brought about by the transmission of sound through the Eustachian tube, and to be necessary before the nerve of the ear will receive upon its outstretched lamina the vibrations that pass through the meatus auditorius, is most difficult to be roused into action; the sound falls upon the ear, but the power which renders it useful is dormant—it sleeps at its post. The sense of touch is equally lost; the insensibility is absolute, it almost approaches to paralysis; the movements become mechanical, the motionless state of the whole body seems almost calculated to inspire a degree of distress in those who behold, whilst the features maintain an inflexible look of solemnity and sorrow.

This condition is observed by the friends without their feeling more than a vague notion that something is going on not quite right, but little are they aware of the possibility of a crisis coming on. Indeed, the general notions of the precursory symptoms of insanity are very much at variance with these signs, and hence it is that they have generally attracted so little regard, and that when any untoward event has occurred that surprise has been excited. The only corporeal malady which, under these circumstances, is complained of, is, that they feel an uncomfortable sensation in the head, and a sort of idea that they are dreaming. They are conscious that they are struggling against some impression that has taken possession of them, yet do they not give vent to others of the complaints they have to make where this occurs; where they once confide to another the feelings they experience, they rarely rush upon their career. It is where they only brood over the thought that has possession of them, that in one instant every power of self-control is lost; the excitation completely overwhelms the reasoning power, the phenomenon of mental alienation is exhibited in its worst form. In the public institutions, it is now not unusual to find persons who have voluntarily placed themselves there, in consequence of having learnt that relief is to be afforded in such cases. At the Biçetre, there was an honest shoemaker, the father of a family, who sought an asylum for himself. He had an open, frank countenance; nothing could be read in it of the horrible idea that had possession of him, which was an impulse to kill his wife and two children, of whom he was very fond. He felt the inclination come on frequently, and was compelled to throw away all the implements of his trade that might assist his madness. Another, who frequently attempted suicide, sought the same relief. The general appearance of these when they first come into a hospital under these circumstances, is that of perfect helplessness: in their worst state, they remain immovable; if their limbs are moved, they remain in the same state almost as in catalepsy; their eyes are wide open, fixed; the physiognomy without expression, the indifference for every surrounding object complete; questions meet with no answers, sometimes they seem not to be heard, at others not to be understood; if a reply is made, it is short, slow, interrupted by a long-continued silence. The will seems to be altogether suspended; none of the attentions to the common wants of life are paid; everything is done in a mechanical manner; there is no inclination to rise, to walk, or even to eat, if this were not carefully watched, and if no assistance were given, there would be no meals taken. These are the external signs of that stupidity which in its worst state exists, and which would have become irresistible madness. These cases are very difficult of cure, and the prognosis is by no means easy. Some have imagined that when there is an alternation of activity and stupidity, there is greater probability of cure; in general, however, where there is no cessation of melancholy, as was the case with Henriette Cornier, there is much more to be dreaded; the automatic performance of the duties of life may be continued, whilst, within, the moral changes are going forward which lead to the outbreak. Whatever may be the ordinary ideas of madness, the flushed face, the rapid and violent gesture, and the maniacal expressions, they are, throughout the whole incubation of the disease—nay, even during the fearful paroxysm—totally absent; the calmness and composure of stupidity being evinced at the moment of executing crime. Who can read the case to which allusions are made, without drawing a lesson from the description given of Henriette Cormer's unembarrassed and cool behaviour.

The real nature of the stupidity, then, in which insane persons are completely involved, would appear to be, that they live in a decided state of hallucination, during which they are isolated from the rest of the world; external impressions have undergone such a complete transformation that in them appears to exist an imaginary world. What can be more striking than the characters grouped together by M. Baillarger, which he has found to exist amongst these unfortunate beings? "The stupid, insane individual," he observes, "is a prey to illusions and hallucinations the most terrific. He is in a desert, in the galleys, in a house of prostitution, in a foreign land, in a prison; an establishment of baths is hell; he takes the mark of a blister for the felon's brand; other insane persons, his fellow-sufferers, for resuscitated dead people, for prostitutes, for soldiers disguised; the forms he sees are hideous, they menace him; the whole world seems drunk; he perceives around him carriages covered with dead bodies; his brother in the midst of torture; a dark ghost at the foot of his bed; the craters of a volcano; abysses without end, prepared to swallow him up; subterranean caves. From all these issue words, and he hears nothing but "You must kill them—you must burn them!" They say opprobrious things to him; his head is full of the noises of clocks and of bells; there are detonations of fire-arms; his parents are fighting with assassins; they are imploring assistance, which he cannot render. He is interrogated about all the actions of his past life; he answers. He hears an instrument at work which is torturing children; his body is traversed with balls; his blood flows upon the ground; he has something on his chest which strangles him; he accuses himself of all his misfortunes. Most frequently he understands the questions that are put to him, but he cannot tell why he does not reply to them—why he does not cry out in the midst of the imaginary dangers which surround him. What is it that restrains his will?—what is it that paralyzes his voice, his limbs? He knows not. Sometimes he has a great desire to call out, to lift himself up, but he cannot. When this condition ceases, he draws a deep sigh, and seems to come out of a deep trance. He asks where he has been—what he has been about. He cannot better compare his situation than by saying that he was in a dream." These are the observations which, during long experience, from the days of Esquirol up to the present moment, M. Baillarger has drawn from conversations with those who have evinced during the state of insanity the most complete stupidity. However incomplete may yet be the knowledge of the manner in which a fixed idea takes possession of the mind at some period to exhibit itself by an act of madness which completely overpowers the will, yet in the vast number of instances it will be found that upon questioning the insane in general, there is a predominant thought that is superior to all the others. It is seldom that the attention of medical men has been directed to this point. Their investigations have not been complete. They have described to us the hallucination surrounded by so many accessory circumstances, that we have not reached the most essential character. This arises from our more commonly having to observe cases where the impressions are transitory and evanescent; and, therefore, we have not learnt to appreciate the importance of the study of that hallucination which is fixed upon one single idea. The particular state of mind in which this fixed idea exists is, under ordinary circumstances, sometimes allied with an irresistible impulse leading to uncontrollable actions: thus, in the Hospital of the Biçetre, there was under the treatment of Dr. Voisin a patient who was under the impression that he was guided entirely by a power whom he called his sovereign. She exercised over him the most absolute sway; not only was she the primary cause of everything that had occurred to him, but she regulated his most minute action, even to his inmost thoughts. He was nothing of himself, but everything was his sovereign. When she paid him a visit, which was principally during the night, he heard her speak, he was conscious of her presence in his body; he knew it, he said, by certain sensations, by certain sufferings, which he experienced, sometimes in one place, sometimes in another. He had never seen her. He had constantly the word "sovereign" in his mouth; and his comrades in the hospital nicknamed him Sovereign. Towards the end of December, and up to the first week of February, he seemed to renounce his erroneous convictions, and he was looked upon as cured. He was the first to laugh at the idea of his sovereign. He acknowledged that the thought was foolish, that he had been in a dream, and wondered at his simplicity in putting faith in it. The 7th of February, he had a relapse. In fact, any one looking at him from the foot of the bed when he was quiet, could see in a moment that a complete change had come over him: his countenance was more animated than usual; his eyes brilliant and moist; the nose especially wore that red hue, which is so commonly visible in the drunkard; the pulse was in its normal state; the functions of the bowels were duly performed; the tongue, however, was white, and slightly furred. Scarcely was he spoken to, when he burst out with the utmost volubility, complaining of the attendants, of his neighbours, of all the world. His speech was incoherent; his lips and a portion of his face agitated with convulsive movements, the muscles scarcely seeming to remain for a moment tranquil; the maniacal excitement was evident. Nevertheless, a slight remonstrance on the part of Dr. Moreau, who saw him, was sufficient to make him quiet, and to render him reserved. He listened and answered to the following questions that were put to him with great composure:—

"Oh, my poor friend! what, have you again returned to your former extravagances? Have you received a new visit from your sovereign?"

"My dear Doctor Moreau, these are not extravagances; it is very true that I have not perceived her presence a long time; but last night she returned to me whilst I was asleep, and awoke me. She compelled me to speak,—to say a vast number of things, of which I understood nothing; she insisted on my whistling and singing."

"All that you say is very absurd. You have had a dream, that is all. How can it be that what you call your sovereign has compelled you to speak and to sing in spite of yourself?—it is an utter impossibility."

"My dear Doctor Moreau, it was by moving about my tongue, that she obliged me, whether I would or not, to speak."

"You have forgotten that I made you hold your tongue, that I had even driven her out of your body, and that I threatened to cut into your side and take her out."

"I assure you, Doctor, that the sovereign told me that it was all the same to her, and that this time she would not stir for all that?"

"Where is she at this moment?"

"Why, Doctor, she is in my head."

"Is she speaking to you now? Listen with attention."

He said, with a smile, he knew very well that she was in his head, but she was determined not to speak.

"Listen, now, again; probably she may make up her mind to speak."

With another smile, "The sovereign has decided not to speak."

"It is evident," says Dr. Moreau, in making his comments on this case, "that the general disorder of the faculties, and a dissociation of ideas, preceded the false conviction and the hallucination. It is not less certain," says he, "according to my opinion, that the last phenomena were the result of the preceding ones. He feels himself irresistibly compelled to speak, to sing, to do actions which he would not do; he is engrossed with the influence of an invisible and mysterious being; and hence his idea of the sovereign. Besides this, convinced that he is made to speak in spite of himself, he without difficulty draws the conclusion that the mysterious being which is within him speaks on her own account. To her he attributes his own thoughts: he speaks aloud his thoughts, and takes these loudly expressed thoughts for words addressed by another to himself." In looking over the descriptions and monographs of hallucinations, it will be found that the fixed idea has gradually sprung up in the midst of illusions and incoherency of thought, that at last, in a vast number of instances, it has a complete ascendancy over all the faculties of the mind, and that then, whenever the maniacal paroxysm comes on, it is irresistibly developed, whilst all the symptoms of violent delirium are present. It is by some pscychologists alleged that there then exists a cerebral congestion, that it is under this physical influence there is an irresistible impulse which urges on to bloodthirstiness, and that this only marks the necessity of depletion. It is an opinion amongst many physicians, that there never can exist a state of disease of the mind without a corresponding disease of organization; and that there will be found some alteration in the structure to account for the presence of the irresistible impulse. Important as is the elucidation of this doctrine, and however we may be allowed, from the analogy which exists in pathological observations, still our present means of arriving at truth are too limited, our anatomical investigations too few, and our intimate knowledge of the brain itself too uncertain, to lead us to any just conclusions. But the field is too important to be neglected; the example of M. Lelut has yet to be followed upon a large scale.

